# Prevalence and Clinicopathologic Characteristics of the Molecular Subtypes in Malignant Glioma: A Multi-Institutional Analysis of 941 Cases

**DOI:** 10.1371/journal.pone.0094871

**Published:** 2014-04-22

**Authors:** Ning Lin, Wei Yan, Kaiming Gao, Yinyi Wang, Junxia Zhang, Yongping You

**Affiliations:** Department of Neurosurgery, The First Affiliated Hospital of Nanjing Medical University, Nanjing, China; Beijing Tiantan Hospital, Capital Medical University, China

## Abstract

**Background:**

Glioblastoma can be classified into four distinct molecular subtypes (Proneural, Neural, Classical and Mesenchymal), based on gene expression profiling. This study aimed to investigate the prevalence, clinicopathologic features and overall survival (OS) of the four molecular subtypes among all malignant gliomas.

**Methods:**

A total of 941 gene expression arrays with clinical data were obtained from the Rembrandt, GSE16011 and CGGA datasets. Molecular subtypes were predicted with a prediction analysis of microarray.

**Results:**

Among 941 malignant gliomas, 32.73% were Proneural, 15.09% Neural, 19.77% Classical and 32.41% Mesenchymal. The Proneural and Neural subtypes occurred largely in low-grade gliomas, while the Classical and Mesenchymal subtypes were more frequent in high-grade gliomas. A survival analysis showed that the Proneural subtype displayed a good prognosis, Neural had an intermediate correlation with overall survival, Mesenchymal had a worse prognosis than Neural, and Classical had the worst clinical outcome. Furthermore, oligodendrocytomas were preferentially assigned to the Proneural subtype, while the Mesenchymal subtype included a higher percentage of astrocytomas, compared with oligodendrocytomas. Additionally, nearly all classical gliomas harbored EGFR amplifications. Classical anaplastic gliomas have similar clinical outcomes as their glioblastoma counterparts and should be treated more aggressively.

**Conclusions:**

Molecular subtypes exist stably in all histological malignant gliomas subtypes. This could be an important improvement to histological diagnoses for both prognosis evaluations and clinical outcome predictions.

## Introduction

Glioma is the most common brain tumor type and an important cause of cancer mortality in adults and children [1], [2]. Biotherapy and molecular-targeted therapies are thought to be future glioma therapy breakthroughs [3]. However, the current grading system based on histopathological diagnoses cannot provide the necessary details for biotherapy and molecular-targeted therapies and have been associated with significant intraobserver variability. Moreover, the underlying etiology of glioma development is unclear. The urgent need for an objective, molecular-based glioma classification system is highlighted by the high rate of divergent diagnoses, inexact prognostic capabilities, and poor therapeutic predictive properties that are based on the current histopathological classification schemes [4]. A molecular classification based on gene profiles could offer an objective subtype-dividing system and indicate subtype or even patient-specific targets for biotherapy and molecular-targeted therapies [5]. Previously, the TCGA network described a robust gene expression-based molecular classification of glioblastomas into Proneural, Neural, Classical, and Mesenchymal subtypes, which are now widely accepted by clinicians and researchers [6].

In the present study, we reviewed 941 glioma samples with gene profiles from three glioma genome databases (CGGA, Rembrandt and GSE16011). Molecular subtypes were assigned by Prediction Analysis for Microarrays (PAM), using the TCGA 840-gene classifier [6]. Furthermore, the prevalence, clinicopathologic features and OS associated with gliomas were investigated according to the molecular subtypes.

## Materials and Methods

### Microarray Data and Analysis of Microarray Gene Expression Data

Microarray data from CGGA (http://www.cgga.org.cn/portal.php), GSE16011 (http://www.ncbi.nlm.nih.gov/geo/) and the Rembrandt databases (https://caintegrator.nci.nih.gov/rembrandt/) were gathered from published studies [5], [7], [8]. The CEL files for GSE16011 and the Rembrandt data set (Affymetrix GeneChip Human Genome U133 Plus 2.0 Array) were separately merged and computed with Matlab software. The expression data were normalized according to theRobust Multi-array Average (RMA) normalization and expressed in a natural scale. A microarray analysis of CGGA glioma samples was performed with the Agilent Whole Human Genome Array, according to the manufacturer’s instructions. Data were acquired on the Agilent G2565BA Microarray Scanner System, with Agilent Feature Extraction Software (v9.1). Probe intensities were normalized with GeneSpring GX 11.0.

### Subtype Prediction and Survival Analysis

Prediction Analysis for Microarrays (PAM) was performed to predict the molecular subtypes of glioma samples from gene profiles, using the Verhaak et al. 840-gene classifier [6], [9]. Kaplan-Meier survival analysis was used to estimate the survival distributions [9]. The log-rank test was used to assess the statistical significance between stratified survival groups with GraphPad Prism 6 statistical software.

## Results

### Prevalence and Clinical Features of Molecular Subtypes in Glioma

**Figure 1:**
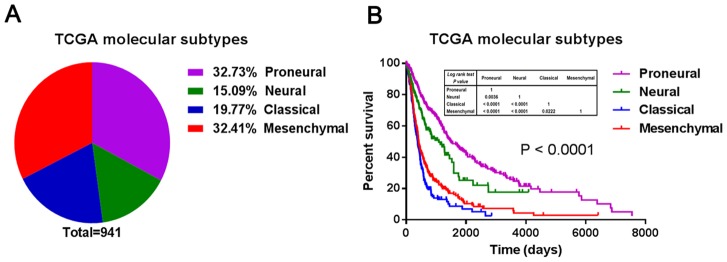
The prevalence and clinical features of the molecular subtypes in all malignant gliomas. (A) Distribution of the TCGA molecular subtypes in all malignant gliomas; (B) survival analysis according to the TCGA molecular subtypes in all malignant gliomas.

A total of 941 samples with gene profiles from incident cases of glioma were gathered from 3 databases (the Rembrandt, GSE16011 and CGGA datasets). The molecular subtypes were predicted with PAM [Figure S1]. As shown in Figure 1A, of the 941 gliomas, 32.73% were Proneural, 15.09% Neural, 19.77% Classical and 32.41% Mesenchymal. Furthermore, a survival analysis of the four subtypes demonstrated that Proneural displayed a good prognosis, while Neural had an intermediate correlation with overall survival and Classical and Mesenchymal showed the worst clinical outcomes [Figure 1B].

**Figure 2:**
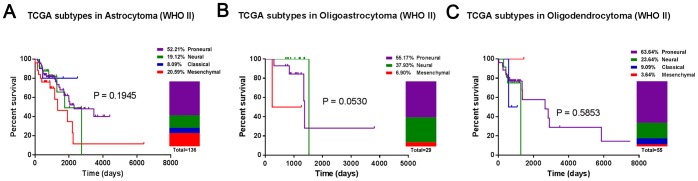
The prevalence and clinical features of the molecular subtypes in low-grade gliomas. (A) astrocytomas, (B) oligoastrocytomas and (C) oligodendrocytomas.

**Figure 3:**
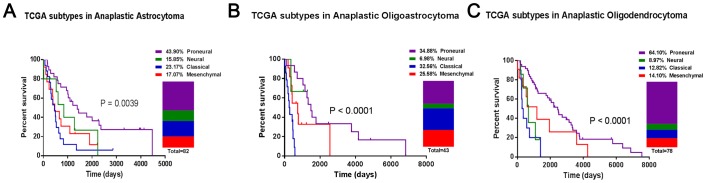
The prevalence and clinical features of molecular subtypes in anaplastic gliomas. (A) astrocytomas, (B) oligoastrocytomas and (C) oligodendrocytomas.

**Figure 4:**
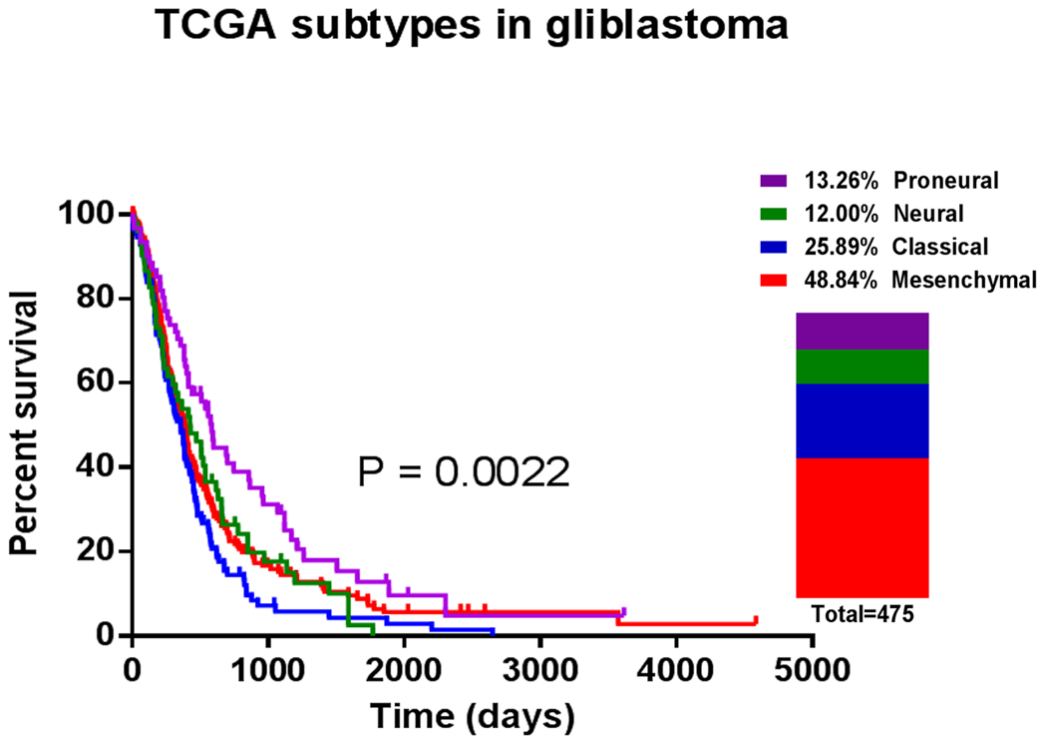
The prevalence and clinical features of the molecular subtypes in glioblastomas.

The prevalence and clinical features of the molecular subtypes were further evaluated in each histological glioma subtype. Only samples with precise histological grade and follow-ups were included in the following analysis. As shown in Figure 2 and 3, Proneural was largely observed in low-grade and anaplastic gliomas. Classical and Mesenchymal accounted for as many as 74.73% of glioblastomas [Figure 4]. Neural was evenly distributed throughout each histological glioma subtype.

Additionally, oligodendrocytomas had a higher prevalence of Proneural(63.64% versus 52.21% for low-grade gliomas; 64.10% versus 43.90% for anaplastic gliomas) and a lower prevalence of Mesenchymal (3.64% versus 20.59% for low-grade gliomas; 14.10% versus 17.07% for anaplastic gliomas), compared with astrocytomas [Figure 2A, 2C, 3A and 3C].

Furthermore, the survival analysis showed that molecular subtypes did not correlate strongly with overall survival. The molecular subtypes significantly stratified the anaplastic gliomas into different prognostic subgroups. In glioblastomas, Proneural had a good prognosis, Classical had the worst clinical outcome and Neural and Mesenchymal had intermediate correlations with overall survival [Figure 4]. Besides, the IDH1 mutation information is available in CGGA and GSE16011 dataset. Through analyzing, the percentage of IDH1 mutation is 73.9%, 43.1%, 22.0% and 22.6% for Proneural, Neural, Classical and Mesenchymal samples of all histological gliomas, respectively.

### EGFR Amplification is a Diagnostic Marker and WHO Grading could not Indicate its Prognostic Value in High-grade Classical Gliomas

**Figure 5:**
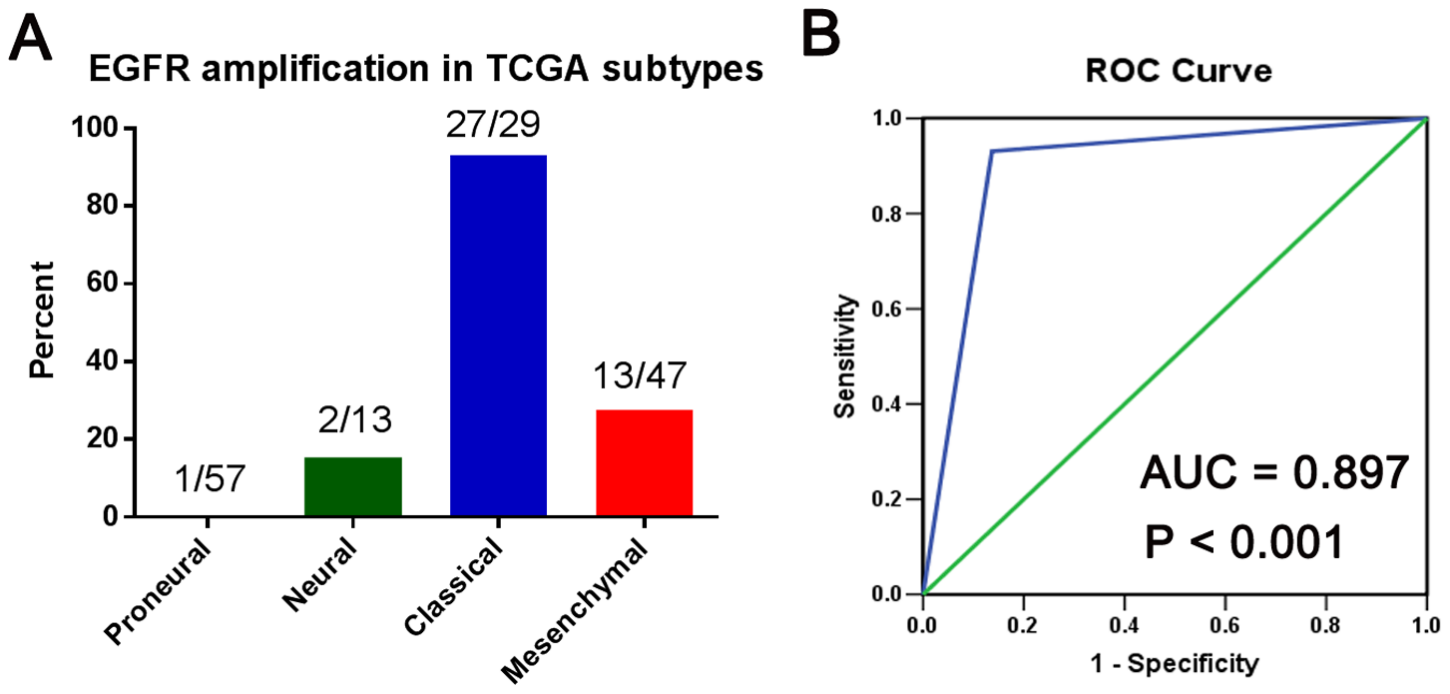
The prevalence of EGFR amplification in the molecular subtypes of malignant gliomas (A) and the ROC curve of EGFR amplification as a potential diagnostic marker of Classical gliomas (B).

Classical glioblastomas are reportedly characterized by EGFR amplifications. In the present study, we first report that EGFR amplification is also enriched in Classical anaplastic glioma samples. As shown in Figure 5A, nearly all Classical gliomas harbored EGFR amplifications. The ROC curve showed that EGFR amplification is a potential diagnostic marker of Classical gliomas (ROC: AUC = 0.897, P<0.001;Figure 5B).

**Figure 6:**
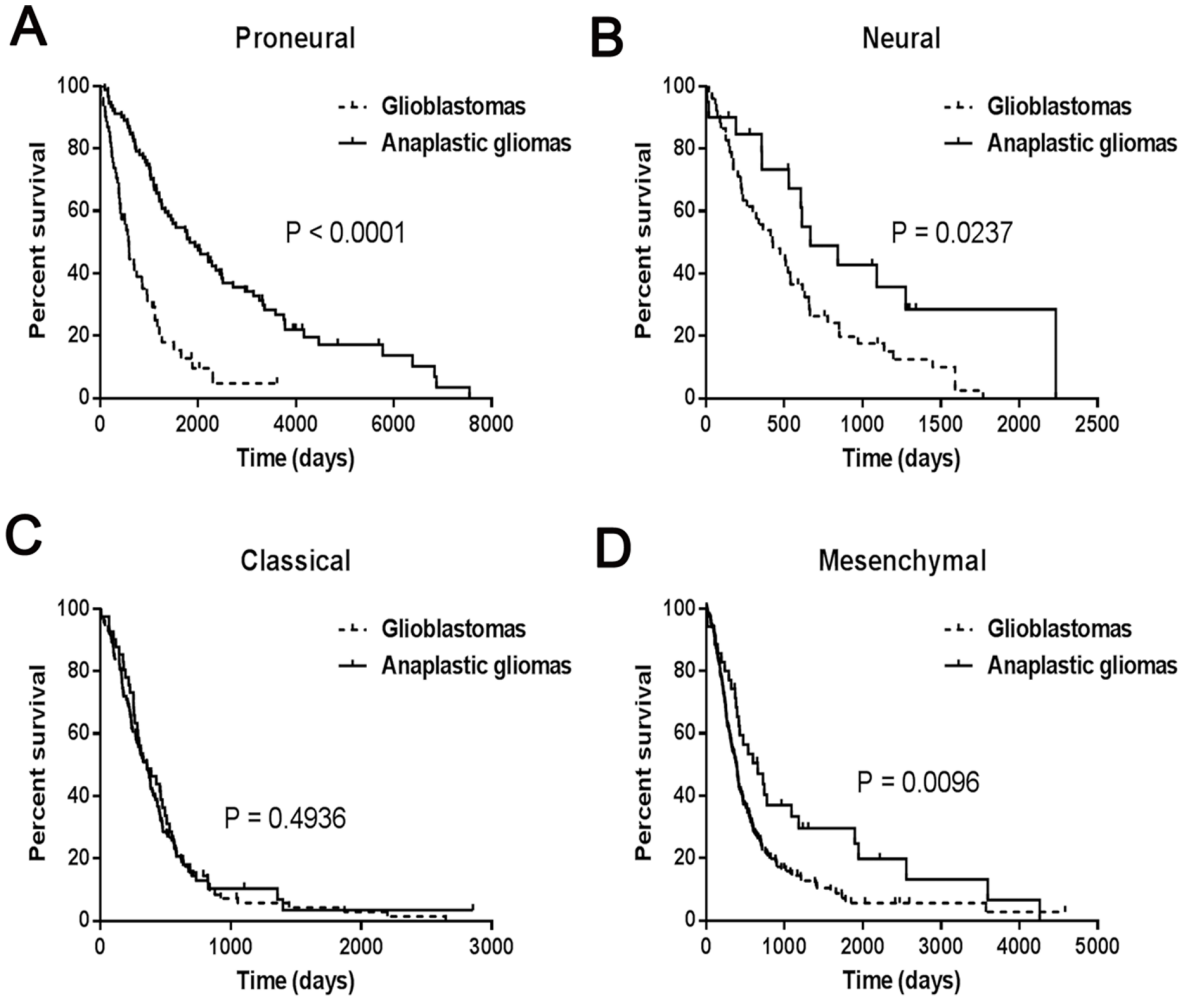
Survival analysis according to the histology and tumor molecular subtypes in high-grade gliomas.

As shown in Figure 6, Classical anaplastic gliomas have similar clinical outcomes as their glioblastoma counterparts and should be treated more aggressively. These findings indicated that the prognostic value of WHO histology grading was not evident in high-grade Classical gliomas.

## Discussion

Gliomas are the most common primary malignant brain tumors in adults, with much heterogeneity in both histopathology and clinical courses [1], [2], [10]. The present grading system for gliomas, which is based on histopathological diagnoses, cannot provide sufficient details for patient-specific biotherapy and molecularly targeted therapies and is associated with significant intra-observer variability. Thus, a glioma classification system based on genetic expression profiles could offer an objective means with which to identify subtype or patient- specific therapeutic targets for biotherapy and molecularly targeted therapies. Glioblastoma, the most lethal type of glioma, has been classified into four distinct molecular subtypes (Proneural, Neural, Classical and Mesenchymal) based on gene expression profiling [6]. In the present study, we evaluated the relevance of the known glioblastoma gene-expression based subtypes to low-grade and anaplastic gliomas. Furthermore, the prevalence, clinicopathologic features and overall survival associated with the four molecular malignant glioma subtypes were investigated.

Reportedly, the intrinsic gene expression profiles of gliomas are better predictors of survival than histology [5]. Several research groups have attempted to subtype gliomas based on gene expression profiling. Phillips et al. defined three subtypes, Mesenchymal, Proneural and Proliferative, in a molecular profile of several high-grade glioma samples [11]. Li et al. used an unsupervised clustering method to identify two main subtypes, defined as GBM-rich and Oligodendroglioma-rich [12]. The TCGA network describes a robust gene expression-based molecular glioblastoma classification that divides cases into the Proneural, Neural, Classical, and Mesenchymal subtypes [6]. Of these classifications, the glioblastoma gene-expression based subtypes submitted by TCGA have been widely accepted. Herein, we collected 941 gene expression arrays with clinical data from the Rembrandt, GSE16011 and CGGA datasets [5], [7], [8]. The molecular subtypes were predicted with PAM. Of these 941 gliomas, 32.73% were Proneural, 15.09% Neural, 19.77% Classical and 32.41% Mesenchymal. The Proneural and Neural subtypes were found largely in low-grade gliomas, while Classical and Mesenchymal were more frequent in high-grade gliomas. A survival analysis showed that Proneural displayed a good prognosis, Neural had an intermediate correlation with overall survival, Mesenchymal had a worse prognosis than Neural, and Classical had the worst clinical outcomes. However, the molecular subtypes have a poor correlation with prognosis in low grade gliomas. Fewer samples in low grade gliomas, bias from loss to Follow-Up and low percentage of Mesenchymal and Classical samples may be the underlying causes of the poor correlation of molecular subtypes and prognosis in low grade gliomas. These results indicated that the glioblastoma gene-expression based subtypes as submitted by TCGA exist stably in other histological glioma subtypes and act as prognostic indicators.

In glioblastoma, the Proneural class was highly enriched for the oligodendrocytic signature, but not the astrocytic signature, whereas the Mesenchymal class strongly associated with the cultured astroglial signature [6]. Our results indicated that oligodendrocytomas were preferentially assigned to the Proneural subtype, while the Mesenchymal subtype included a higher percentage of astrocytomas, compared with oligodendrocytomas. Additionally, EGFR amplification was enriched in Classical glioblastomas. In the present study, we found that nearly all Classical samples, including glioblastomas and anaplastic gliomas, harbored EGFR amplifications; also, Classical anaplastic gliomas had similar clinical outcomes to their glioblastoma counterparts and therefore should be treated more aggressively.

In summary, the molecular glioblastoma subtypes suggested by the TCGA network are relevant for low-grade and anaplastic gliomas and are associated with different prognoses. The above-described molecular subtyping system could be an important improvement to routine histological diagnoses and might guide therapeutic glioma management.

## Supporting Information

Figure S1 **The molecular subtypes of glioma samples from GSE16011 and Rembrandt datasets were predicted using Prediction Analysis for Microarrays (PAM).**
(TIF)
