# Supplementary figures and images for: Prevalence and Clinicopathologic Characteristics of the Molecular Subtypes in Malignant Glioma: A Multi-Institutional Analysis of 941 Cases
Source: PLoS One. 2014 Apr 22;9(4):e94871. doi: 10.1371/journal.pone.0094871 (PMC3995672; doi:10.1371/journal.pone.0094871)

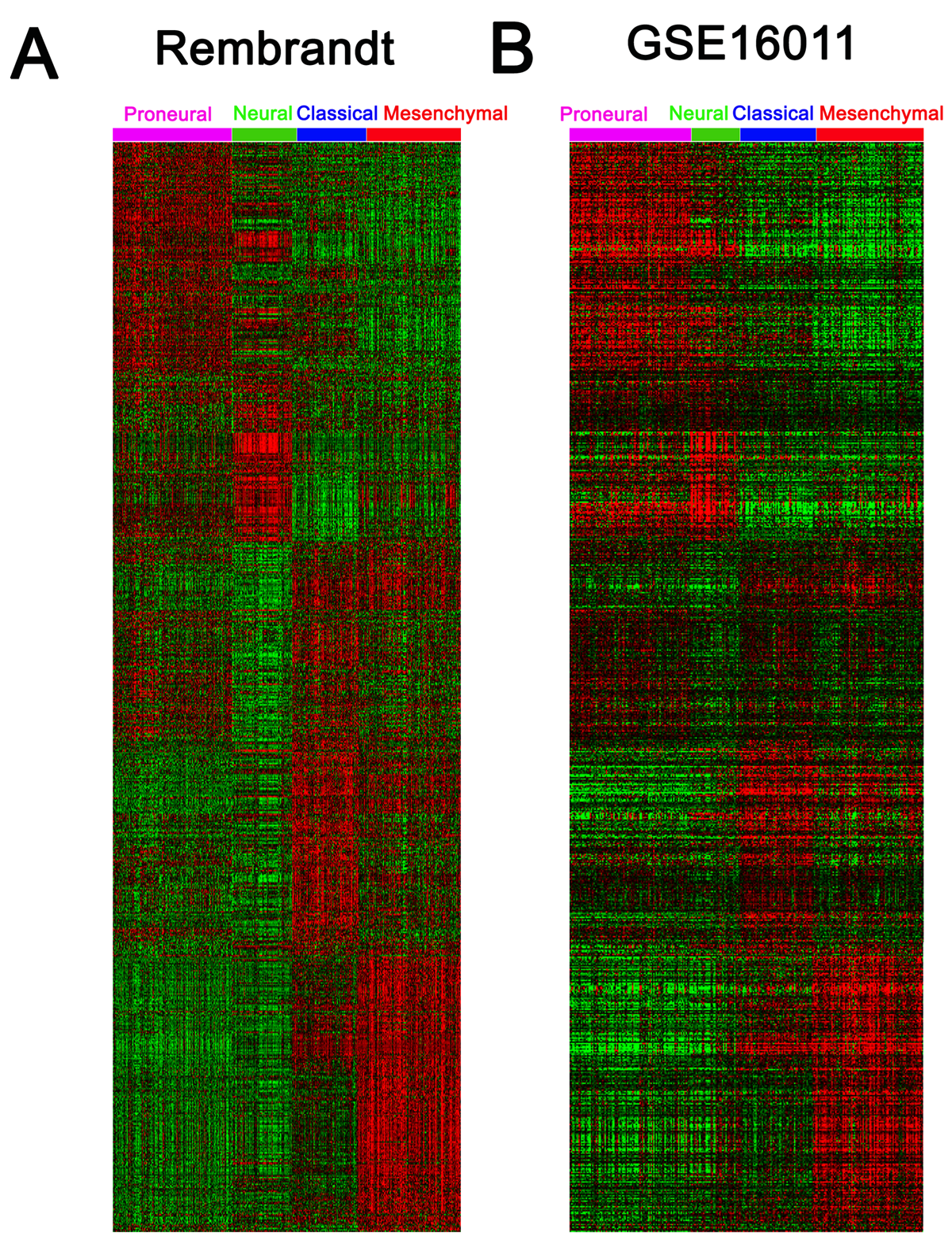

Supplement: Figure S1 — The molecular subtypes of glioma samples from GSE16011 and Rembrandt datasets were predicted using Prediction Analysis for Microarrays (PAM). (TIF) [file pone.0094871.s001.tif]
